# Beyond morphology: imaging the glioblastoma microenvironment in the era of quantitative neuro-oncology

**DOI:** 10.3389/fmed.2026.1846372

**Published:** 2026-07-17

**Authors:** Lorenzo Arena, Giuseppe Espa, Giovanni Bertalot, Paolo Soda, Orazio Caffo, Dylan J. H. A. Henssen, Paola Feraco

**Affiliations:** 1 Centre for Medical Sciences (CISMed), University of Trento, Trento, Italy; 2 Department of Economics and Management, University of Trento, Trento, Italy; 3 Unit of Artificial Intelligence and Computer Systems, Department of Engineering, Università Campus Bio-Medico di Roma, Roma, Italy; 4 Department of Nuclear Medicine, University Hospital Leipzig, Leipzig, Germany

**Keywords:** glioblastoma, MRI, neuroncology, PET, radiomics, tumor microenvironment

## Abstract

Glioblastoma (GBM) is the most aggressive primary brain tumor in adults and is characterized by rapid progression, marked spatial and molecular heterogeneity, and poor prognosis despite multimodal treatment strategies. Tumor behavior is not determined solely by intrinsic genetic alterations but also by dynamic interactions within the tumor microenvironment, including hypoxia, aberrant angiogenesis, immune modulation, and metabolic reprogramming, which are major drivers of treatment resistance and disease recurrence. Magnetic resonance imaging (MRI) remains the cornerstone of diagnosis and treatment planning; advanced quantitative techniques have expanded its role beyond structural assessment, enabling *in vivo* characterization of tissue cellularity, vascular architecture, perfusion, and microenvironmental dynamics. Positron Emission Tomography (PET) provides complementary metabolic and molecular information, improving tumor delineation, detection of infiltrative disease, and assessment of hypoxia and treatment response. PET-MRI integration within a multimodal framework enables spatially resolved mapping of tumor heterogeneity and microenvironmental niches that cannot be adequately assessed by either modality alone. Radiomics and radiogenomics approaches further enhance this paradigm by extracting quantitative imaging features that reflect underlying biological processes and linking imaging phenotypes with molecular and clinical outcomes. Artificial Intelligence enables automated feature extraction, multimodal data integration, and predictive modeling for diagnosis, prognosis, and treatment response assessment. Despite these advances, clinical translation remains limited by methodological heterogeneity, lack of standardized acquisition protocols, and insufficient prospective validation. Future research should prioritize harmonized multicenter studies and biologically informed multimodal analytical frameworks to enable the routine implementation of microenvironment-oriented precision imaging in GBM management.

## 1 Introduction

Diffuse gliomas account for approximately 80–85% of all malignant primary brain tumors, with an estimated annual incidence of about 7 cases per 100,000 individuals (1). Among these, GBM represents the most aggressive and lethal entity in adults, with a median overall survival ranging from 15 months to 2 years despite the current standard of care based on maximal safe resection followed by temozolomide and radiotherapy (2). This poor prognosis is largely driven by the tumor’s highly infiltrative growth pattern and profound intratumoral molecular and phenotypic heterogeneity, features that compromise therapeutic efficacy, promote treatment resistance, and limit the effectiveness of conventional strategies (3, 4). The heterogeneous tumor microenvironment (TME) is a complex interplay between malignant and non-malignant components modulating tumor behavior through cytokine signaling and metabolic interactions that play a central role in GBM progression, immune evasion, and therapeutic resistance (5). Hypoxia, aberrant angiogenesis, and immune suppression constitute three fundamental microenvironmental domains sustaining tumor growth and promoting survival of therapy-resistant cell populations, including GBM stem cells (6). The spatially heterogeneous niches (hypoxic, perivascular, and immune-related) are dynamically reprogrammed during treatment, highlighting the limitations of single-site tissue sampling and emphasizing the need for spatially resolved, non-invasive approaches capable of capturing tumor heterogeneity *in vivo* (6).

**Figure 1:**
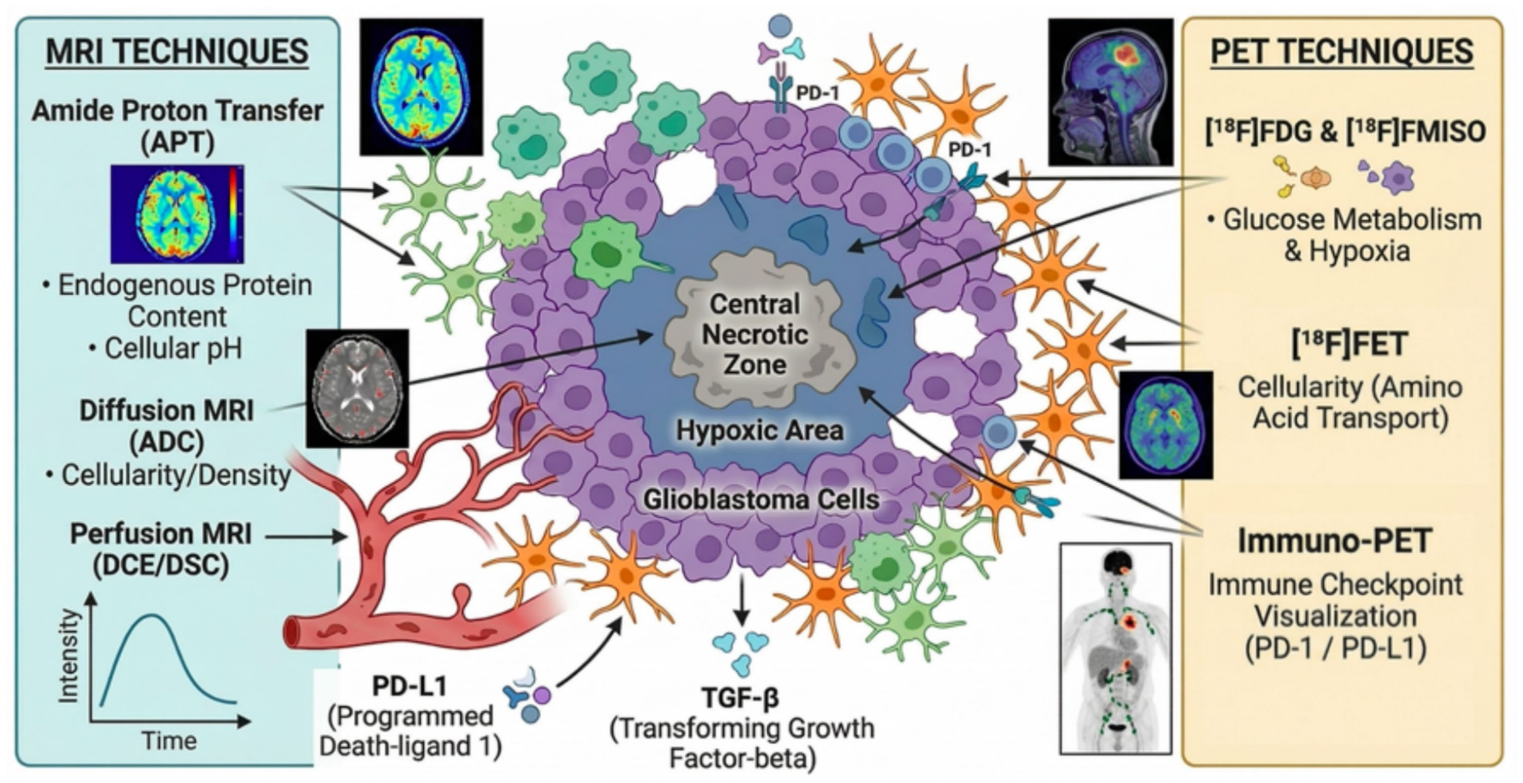
Multimodal imaging of the glioblastoma tumor microenvironment.

Advanced neuroimaging has therefore emerged as essential for characterizing the tumor microenvironment at the whole-tumor level. Multiparametric MRI and PET enable non-invasive assessment of vascular proliferation, cellular density, metabolic activity, and tissue hypoxia (Figure 1) (7–11), providing clinically relevant information for tumor characterization, therapy planning, and longitudinal monitoring within the World Health Organization Central Nervous System classification framework (1). However, the complexity of the GBM microenvironment generates high-dimensional datasets exceeding the interpretative capacity of conventional qualitative analysis. AI and radiomics have emerged as critical frameworks for extracting quantitative features from multimodal imaging data and converting them into reproducible biomarkers of tumor biology (12). Integration of imaging-derived metrics with molecular and immunological information enables predictive models supporting personalized therapeutic strategies (13).

Recent advances in spatial transcriptomics, high-parameter imaging, and AI-driven analytics confirm that immune phenotype, metabolic status, and vascular organization vary significantly across GBM regions and treatment stages (14), reinforcing the concept of the TME as a spatially organized biological system. This narrative review explores advanced quantitative MRI, PET-based radiomics, and multimodal imaging integration, highlighting AI’s contribution to diagnostic precision, risk stratification, and individualized treatment planning in GBM (12, 13).

Schematic overview of key biological components in the GBM microenvironment (e.g., neoangiogenic vessels, necrotic core, hypoxia, and signaling pathways like TGF-*β* and PD-L1) along with their corresponding MRI and PET characterization techniques. The integration of these multimodal imaging approaches enables spatially resolved assessment of tumor heterogeneity to support precision neuro-oncology.

## 2 Methods

This review was designed as a narrative review rather than a systematic review. The literature search was intended to identify the most relevant and representative evidence supporting the major topics discussed, rather than to perform an exhaustive systematic evidence synthesis.

A literature search was conducted in PubMed and PMC databases using the following keyword clusters: (3) MRI radiomics: (“Glioma” OR “Glioblastoma”), “habitat imaging,” “MRI radiomics,” “DWI,” “diffusion weighted,” “perfusion,” “DSC,” “DCE,” “cellularity,” “angiogenesis,” “neovascularization”; (4) PET radiomics: (“glioma” OR “Glioblastoma”), “PET Radiomics,” (“FET” OR “FMISO” OR “FAPI”); (5) Multiparametric integration: (“Glioma” OR “Glioblastoma”), “PET/MRI,” “Radiomics,” “Multiparametric,” “Artificial Intelligence,” “Machine learning.” Boolean operators were used to combine search strings and refine results.

**Figure 2:**
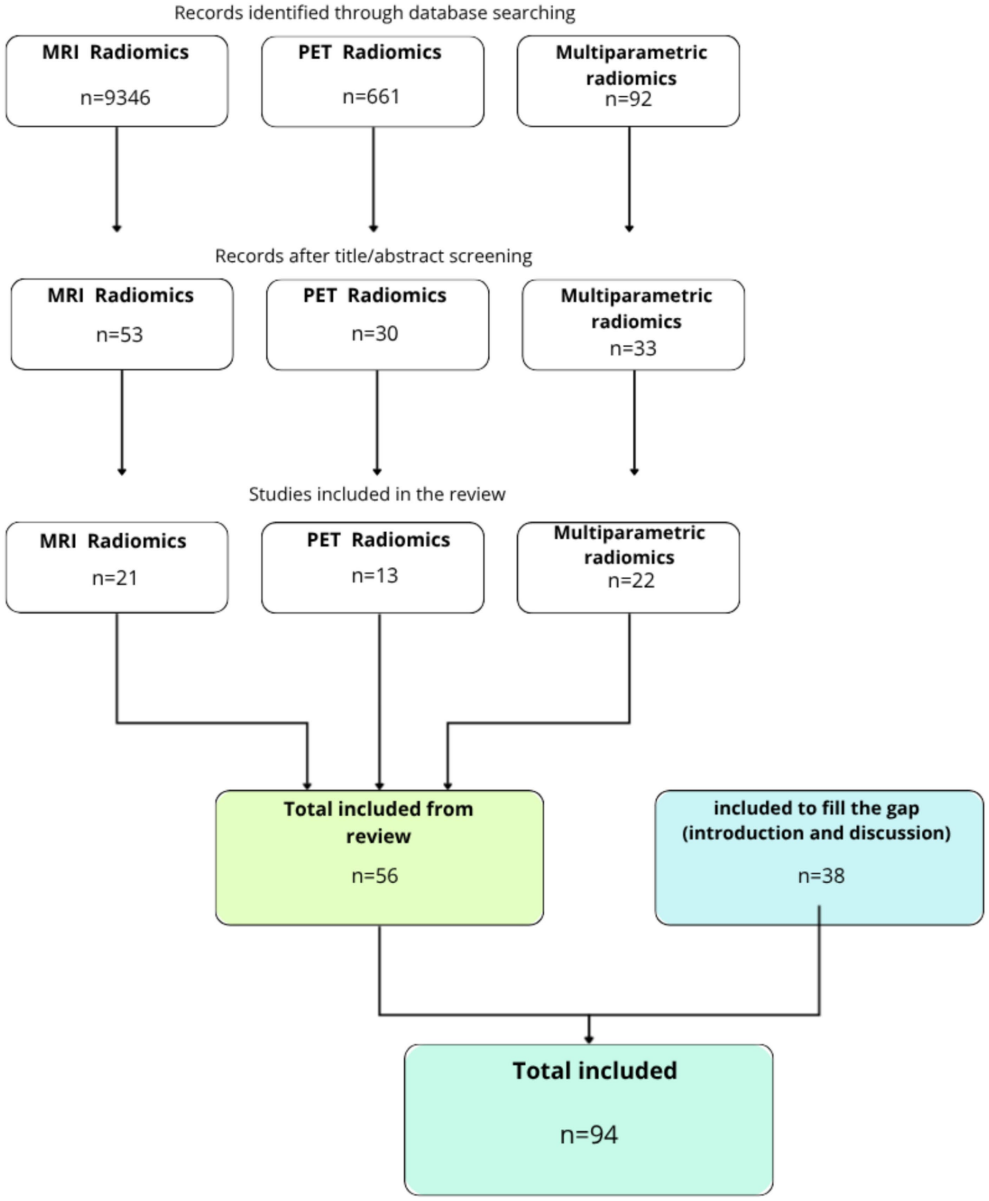
Narrative literature selection workflow. Schematic representation of the study selection workflow, categorized by imaging modality and methodology (MRI radiomics, PET radiomics, and AI/multiparametric approaches). The diagram illustrates the number of records initially identified, screened by title/abstract, and ultimately included in the final narrative review (*n* = 94).

Articles were initially screened on the basis of title and abstract; only full-text systematic reviews and experimental papers in English were included in the research. The most pertinent full-text papers (papers that included original quantitative MRI/PET data, excluding case reports and non-systematic reviews) were subsequently retrieved and analyzed. The primary search was restricted to 2020–2026; methodologically relevant studies published before 2020 were incorporated through backward citation tracking, and targeted PubMed searches addressed residual gaps. The initial search yielded 116 papers, of which 56 met the inclusion criteria; however, after careful exploration of cited papers and a targeted search to fill the gaps in the narrative review, a total of 92 papers were included in this review. Titles, abstracts, and full texts were screened by one author, while article selection and thematic organization were discussed and verified by all co-authors; a flowchart of the selected paper can be accessed in Figure 2.

## 3 MRI-based radiomics and radiogenomics: mapping the tumor microenvironment and molecular landscape

Advanced MRI techniques have expanded glioma characterization beyond structural assessment, enabling comprehensive evaluation of the tumor microenvironment. Perfusion imaging plays a central role: Dynamic Susceptibility Contrast (DSC)-MRI, Dynamic Contrast Enhanced (DCE)-MRI, and Arterial Spin Labeling (ASL) quantify tumor vascularization through relative Cerebral Blood Volume (rCBV) estimation, vascular permeability and blood- brain barrier integrity assessment, and non-contrast cerebral blood flow measurement using magnetically labeled water, respectively (15, 16).

Diffusion Weighted Imaging (DWI) enables Apparent Diffusion Coefficient (ADC) quantification reflecting tissue cellularity and membrane integrity, with lower values associated with densely packed tumor cells and higher values corresponding to necrotic or edematous regions (17, 18). Advanced DWI models, including Diffusion Tensor imaging (DTI) and Diffusion Kurtosis Imaging (DKI), further enhance sensitivity to tissue heterogeneity. MRI-spectroscopy detects metabolic alterations linked to specific molecular subtypes such as isocitrate dehydrogenase (IDH)-mutated gliomas (19), while Amide proton transfer (APT) imaging provides contrast related to mobile proteins without exogenous agents, representing a radiation-free surrogate of cellular metabolic activity comparable to selected PET tracers (20, 21). Although DSC-MRI and DWI remain most widely implemented clinically, the broader multiparametric framework has gained relevance for treatment planning, target delineation, and infiltration mapping (19).

Habitat imaging has emerged as a spatially resolved framework integrating quantitative MRI biomarkers into biologically meaningful tumor subregions. By clustering voxels with similar imaging characteristics, habitat analysis generates maps reflecting glioma microenvironmental architecture with spatial detail unavailable through whole-tumor metrics alone (22, 23). Key methodological considerations include the selection of biomarkers such as ADC and rCBV, alongside standardized registration and k-means clustering algorithms (24). Radiomics enables systematic extraction of high-dimensional imaging features capturing tumor phenotypes beyond visual interpretation (25). In glioma research, radiomic approaches have demonstrated the ability to link imaging features with underlying biological and molecular processes, forming the basis of radiogenomics analysis. Histopathological validation confirms that imaging-derived features reflect genuine biological signals: ADC-based models correlate with tumor-associated macrophage infiltration (Area Under the Curve AUC = 0.768), rCBV correlates with neovascularization markers, and diffusion metrics correlate with T-lymphocyte infiltration (26, 27). Radiomic and habitat-based models have shown promising predictive capabilities, including Isocitrate Dehydrogenase (IDH) genotype prediction with an AUC approaching 0.94, identification of spatial recurrence patterns, and mapping of subregional therapy resistance (28–30). APT-derived metrics enable IDH subtype classification with an AUC of 0.858 (31).

Radiogenomics links quantitative imaging features with molecular and genomic data, enabling non-invasive virtual biopsy of the tumor’s biological profile (32, 33). Both supervised models predicting IDH mutation or *O*-6-methylguanine-DNA methyltransferase (MGMT) methylation and unsupervised approaches identifying imaging-derived phenotypes have been applied (34). IDH prediction shows the most consistent performance, while MGMT correlates remain less reliable due to methodological heterogeneity and limited adherence to frameworks such as TRIPOD (35). APT imaging distinguished MGMT from non-MGMT high-grade gliomas with an AUC of 0.702 (36). Multiparametric MRI phenotypes combining rCBV, ADC, and enhancement patterns explain up to 57.9% of transcriptional variance in GBM (37), while immune indices such as the ICI score represent prognostic benchmarks that advanced imaging may approximate non-invasively (38). In review, Radiogenomics signatures show strong associations between imaging phenotypes and survival (HR 3.68; 95% CI: 2.08–6.52), confirmed in external validation (HR 2.02; 95% CI: 1.19–3.41) (39). Integrative approaches combining radiomic clustering with transcriptomic profiling identified biologically distinct GBM subtypes with poorer outcomes, with transcription factors such as VAX2 (Ventral Anterior Homeobox 2) proposed as drivers of aggressive phenotypes (40). Multi-omics strategies integrating radiomics with transcriptomic and proteomic data have improved patient stratification and identified minimal gene and protein signatures associated with recurrence risk in IDH-mutant gliomas (41). Despite promising predictive performance, these results should be contextualized in their exploratory context; in fact, most of the papers have a sample size smaller than 100 participants (26, 29, 30, 36), with the works exceeding 100 participants doing so by a small margin (28, 31). Although valid, radiomic procedures that include such reduced samples present a high risk of overfitting, high specificity with reduced sensitivity, or vice versa. Most importantly, almost every study included in this review relies on single-center data. Although this choice has some positive sides, such as a reduced necessity of harmonization and an almost identical extraction procedure, on the other hand, it implies the lack of external validation and, by reflection, a reduction in generalization power. For this reason, the results included in this review are good in terms of exploratory value, but a full implementation of such methodologies in everyday clinical practice requires a model trained on a larger dataset and scalable across different centers through standardized definitions and computation protocols for radiomic features aimed to improve cross-study comparability.

## 4 PET imaging: metabolic profiling and tumor delineation

PET provides a unique opportunity to non-invasively characterize biological processes that cannot be adequately captured by MRI alone, detecting coincident annihilation photons from positron-emitting radiotracers to enable *in vivo* quantification of metabolic activity at the tissue level (42). In gliomas, overexpression of L-type Amino Acid Transporter 1 (LAT1) transporters forms the biological basis for amino acid PET imaging, with these tracers demonstrating minimal uptake in normal brain parenchyma and allowing high-contrast tumor delineation, including non-enhancing infiltrative components undetectable on contrast-enhanced MRI (43).

Different PET tracers provide complementary biological information. Commonly used tracers in GBM include 18F-Fluorodeoxyglucose (FDG), 18F-fluorothymidine (FLT), 11C- Methionine (MET), and 11C-acetate (43), while amino acid tracers such as 18F-fluoroethyl-L-tyrosine (FET) and 18F-Fluorodopa (FDOPA) offer high specificity for viable tumor tissue and are widely applied for delineation and recurrence assessment. Hypoxia imaging with 18F-fluoromisonidazole (FMISO) provides a quantitative assessment of tumor oxygenation, and emerging tracers such as FAPI allow direct evaluation of the stromal compartment (43). FMISO-PET has demonstrated performance comparable to MRI in predicting one-year survival (AUC 0.870), with further improvement in integrated PET-MRI models (44).

From a clinical perspective, amino acid PET provides substantial added value for tumor delineation and treatment response assessment. FET-PET identifies tumor volumes extending beyond FLAIR-defined abnormalities in up to one-third of patients (45) and distinguishes astrogliosis from true tumor infiltration, a differentiation challenging with MRI alone (46). The 2023 ESTRO-EANO guidelines formally recommend amino acid tracers (MET, FET, and FDOPA) for radiotherapy target delineation, enabling more accurate target definition and safer dose escalation in re-irradiation planning (47). Radiomics applied to MET-PET outperforms conventional tumor-to-normal uptake ratios in differentiating recurrence from radionecrosis and predicting survival (48, 49).

Beyond tumor delineation, PET provides clinically relevant prognostic information. Hypoxic volume and tissue-to-blood ratio from ^18^F-MISO independently predict time to progression and overall survival (*p* < 0.003) (50). Hypoxia metrics further discriminate pseudoprogression from true recurrence and predict survival in bevacizumab-refractory disease (51, 52). FAPI PET targeting Fibroblast Activation Protein-*α* has emerged as a promising tool for stromal microenvironment assessment, with early studies showing diagnostic performance comparable to DSC-MRI, though prospective validation remains necessary. Immuno-PET enables *in vivo* visualization of immune checkpoints and immune cell trafficking through radiolabeled monoclonal antibodies, with potential applications in patient selection, treatment monitoring, and immunotherapy response prediction (53). PET radiomics represents a rapidly evolving field with substantial potential, though future progress depends on radiotracer expansion, workflow harmonization, and integration with multiparametric MRI and AI-based analytical frameworks (54–57). However, the PET-radiomic literature remains considerably smaller than its MRI-radiomics counterpart, both in volume and in the number of methodologically robust studies. Consequently, definitive conclusions regarding the comparative value of PET-based radiomics approaches should be deferred until further evidence emerges from the literature.

A summary of the main results for each modality is reported in Table 1.

**Table 1:** Methodological and biological comparison of MRI, PET, and hybrid MRI/PET applications in glioblastoma.

Imaging modality	Technique	Biologic target	Clinical application	Level of validation	Main limitation
MRI	Perfusion, diffusion, habitat imaging	Vascularization, cellularity, membrane integrity, microenvironment architecture	rCBV is correlated with vascularization marker; ADC is correlated with tumoral infiltration (26, 27); good discrimination within molecular status groups (36)	Clinically consolidated	Acquisition variability
PET	FDG, FMISO, FAPI, FET/MET, FDOPA	Glucose/aminoacidic metabolism, tumoral hypoxia, stromal areas and immunologic checkpoint	FET-PET identify tumor hypoxic volume and predicts survival (50); diagnostic performance and feature detection almost precise as MRI (43, 45, 61)	Clinically validated, some markers such as FAPI and FMISO valid only for research	Needs expensive technology and tracers
MRI/PET	Simultaneous or sequential acquisition	Multidomain spatial mapping	Increment in accuracy respect modality only approaches (63)	Clinically feasible with good performance	Expensive, scarcity of infrastructures

## 5 Multimodal PET/MRI integration

**Figure 3:**
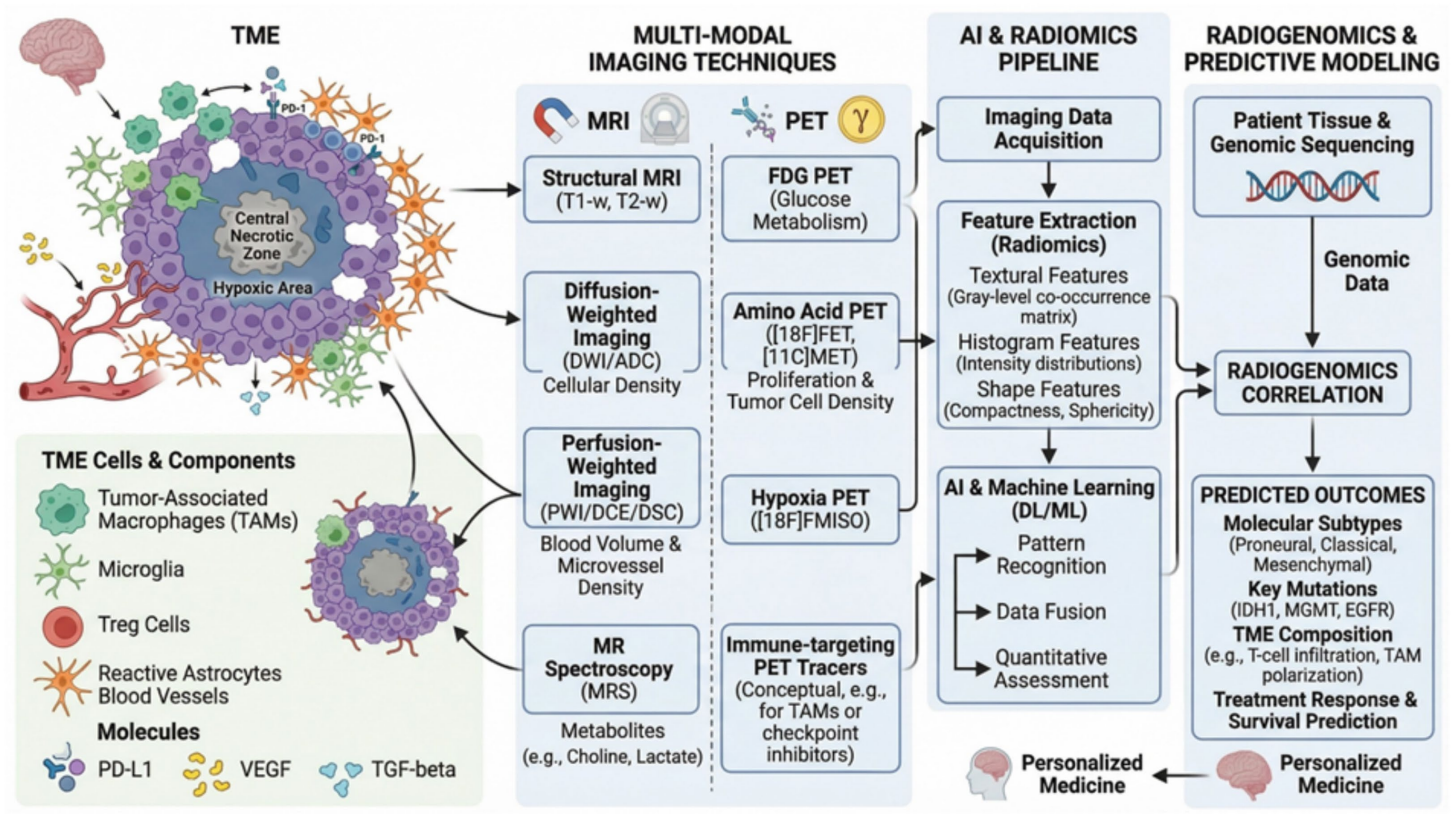
Schematic workflow integrating multiparametric MRI (structural, diffusion, perfusion, and metabolic data) and PET imaging (targeting glucose, amino acids, hypoxia, and immune markers) to characterize the tumor microenvironment. Radiomic features extracted from these modalities are combined via machine learning to predict molecular alterations, tumor subtypes, and clinical outcomes for personalized patient management.

Multimodal PET/MRI integration addresses the intrinsic limitations of each modality when used in isolation by combining structural, perfusion, diffusion, and metabolic information into spatially co-registered maps. This integrated framework enables a more comprehensive characterization of tumor biology and supports clinically meaningful decision-making. Both simultaneous and sequential acquisition protocols have demonstrated comparable diagnostic performance in patients with GBM, supporting the feasibility of multimodal imaging in routine clinical practice (58) (Figure 3).

From a biological perspective, combined PET/MRI analysis provides complementary biomarkers reflecting distinct aspects of tumor physiology. Amino acid PET uptake correlates with tumor cellularity, perfusion metrics such as CBV reflect neovascularization, and diffusion parameters show an inverse relationship with cellular density (26). Simultaneous acquisition of DSC-MRI, 18F-FLT PET, and ^18^F-MISO PET has enabled identification of spatially distinct tumor compartments characterized by proliferative, hyper vascularized, and hypoxic phenotypes extending beyond contrast-enhanced tumor margins (59).

Treatment response assessment represents one of the most clinically impactful applications, particularly in recurrent GBM, where conventional MRI often fails to distinguish tumor progression from treatment-related effects. Disease progression undetected by MRI per RANO criteria has been identified using FET-PET during antiangiogenic therapy (60). FET-PET has demonstrated superior diagnostic accuracy over perfusion MRI alone (AUC = 0.96 vs. 0.83), with combined PET/MRI further improving specificity and risk stratification (61). In patients treated with multi-kinase inhibitors, FET-positive and ADC-derived metabolically active volumes covaried significantly (R = 0.54), both predicting overall survival comparably to RANO criteria (62).

Machine learning techniques have enabled voxel-level multimodal analysis. A supervoxel-based pipeline incorporating FET-PET, CBV-DSC, and contrast-enhanced T1 features achieved diagnostic accuracy approaching 80% and AUC values exceeding 0.85, with most informative features derived from PET (63). These analyses revealed spatially coexisting regions of progression and treatment-related change, underscoring the value of high-resolution multimodal approaches.

Although extremely useful, the integration of PET/MRI has to be taken into consideration with all its limitations. The reviewed literature, in fact, overlooks the substantial economic and infrastructural barriers to clinical implementations: hybrid PET/MRI scans, for example, are less available, require specific profiles of expertise in both systems, and generally require longer and more expensive acquisition procedures, making the simultaneous acquisition paradigm inaccessible to the majority of GBM patients (64). To a lesser extent, sequential PET amino acid tracers face significant access barriers, requiring costly procedures, inconsistently reimbursed by health insurance companies, facing heterogeneous regulatory status of amino acid PET tracers across jurisdictions, and logistic constraints, such as the necessity of an on-site cyclotron for 11C-MET, that are often difficult to overcome (65). However, some machine learning algorithms, for example, federated learning approaches, partially address this problem, limiting the necessity of single centers to possess the full acquisition stack, while synthetic PET generation from MRI could provide metabolic information in settings with limited access to specific PET tracers. The gap, however, still exists, and stress on the implementation must be explicitly prioritized.

## 6 Artificial intelligence for multimodal radiomics

Artificial intelligence (AI) applications in GBM imaging have rapidly expanded, encompassing synthetic data generation, multimodal image fusion, automated segmentation, and radiomic classification. These approaches aim to address fundamental challenges, including data heterogeneity, limited sample size, and the complexity of integrating multiple modalities into clinically actionable models. As highlighted by recent methodological work, the value of multimodal deep learning depends not only on combining different data sources, but also on preserving modality-specific information while learning meaningful interactions across them (66).

One of the most active areas involves data generation and modality fusion. Multimodal fusion networks and generative models have demonstrated the ability to reconstruct missing imaging modalities and generate synthetic datasets, improving robustness and addressing data scarcity (67, 68). Adversarial learning frameworks have enabled classification of high- and low-grade gliomas using latent representations of absent MRI sequences, with AUC values approaching In review This is a provisional file, not the final typeset article 0.88 (69), while GANs have produced realistic synthetic MRI datasets offering solutions to privacy constraints (70, 71). Transformer-GAN architectures have enabled standard-dose PET reconstruction from low-dose PET and MRI, and image-to-image translation models have demonstrated the feasibility of generating synthetic amino acid PET from contrast-enhanced MRI, with adequate performance for glioma grading (AUC approximately 0.81) (72, 73). Fusion strategies should nonetheless be evaluated not only by accuracy, but by their ability to remain informative when modalities are incomplete or unevenly available across centers (66).

Beyond image synthesis, AI has shown strong performance in classification and prognostic modeling. Machine learning algorithms have achieved AUC values approaching 0.98 for tumor classification and predictive accuracy exceeding 98% for survival estimation (74). Models integrating imaging with clinical and molecular variables consistently outperform imaging-only approaches in survival stratification and pseudoprogression detection (75). Convolutional neural networks and hybrid architectures have reached classification accuracies approaching 99% in differentiating intracranial tumor types (70), while U-Net-based and CNN-transformer models have addressed automated segmentation (76), and federated learning enables multicenter training with privacy preservation (77, 78).

A critical dimension of multimodal AI concerns the choice of data fusion strategy; current methods can be broadly categorized based on the step of the analysis at which the modality fusion is introduced. Early fusion introduces an integration of raw features before the model training, intermediate (or joint) fusion that operates at feature-representation level, and late fusion, which combines modality-related prediction when reporting the output. In a recent study on adult and pediatric glioma cohorts, joint fusion of genomics and imaging data outperformed both early and late fusion strategies in terms of prognosis prediction (79); on the other hand, different studies demonstrate that, when dealing with small samples, late fusion shows greater resistance to overfitting when dealing with survival prediction (80). Other fusion methods, based on Attention and tensor, instead, offer enhanced interpretability through dynamic modality contribution weighting (81). Overall, the fusion approaches should be guided by the task characteristics, like sample size, modality heterogeneity and interpretability, to provide an useful tool to study brain tumors characteristics and predict their development; Multimodal studies, however, are far from being systematically implemented, often lacking unimodal comparisons, and infrequently including external validation or explainability analyses (66). Moreover, most of the existing multimodal studies focus on radiomics and genomics; at the moment, there is a gap in the literature for what concerns multimodal PET-MRI studies on Gliomas. For these reasons, despite the advances in the field, most AI-based GBM studies rely almost exclusively on MRI, while PET-derived metabolic information remains underexplored. This gap is particularly relevant given the demonstrated biological complementarity between modalities, and the finding that in one of the best-performing multimodal response assessment pipelines, the majority of informative predictive features were derived from PET rather than MRI (82).

## 7 Discussion and future directions

The evidence reviewed consistently converges on a central conclusion: the biological complexity of GBM cannot be adequately captured by any single imaging modality or analytical framework. Advanced quantitative MRI, particularly when organized through habitat imaging, provides spatially resolved maps with demonstrated histopathological validity (22–27), enabling clinically relevant predictions of molecular status, recurrence patterns, and treatment response (28–31). PET contributes complementary biological information inaccessible to MRI alone, including metabolic quantification (42, 43), improved delineation of infiltrative margins beyond FLAIR abnormalities (45, 46), characterization of tumor hypoxia with independent prognostic value (50–52), and assessment of the stromal microenvironment through emerging tracers such as FAPI (43). Overall, although the available evidence consistently supports the biological relevance of quantitative MRI, PET, radiomics, and AI, the current level of evidence remains predominantly exploratory because most studies are retrospective, single-center investigations with limited external validation.

When integrated within a unified multimodal framework, these techniques enable comprehensive characterization of intratumoral heterogeneity, including spatial coexistence of viable tumor and treatment-related changes at the voxel level (59, 63, 83, 84), patterns difficult to resolve using individual modalities alone (60–62). AI provides the analytical infrastructure to manage this complexity, with multimodal models consistently outperforming unimodal approaches across classification, segmentation, and outcome prediction (68, 70, 74, 75, 77). However, better predictive performance alone is insufficient for clinical adoption: models must also be interpretable, reproducible, and robust to incomplete data (66).

Despite these advances, clinical translation remains constrained by persistent limitations. Substantial methodological heterogeneity across acquisition protocols, tracer availability, habitat clustering strategies, and radiomic pipelines limits cross-study comparability and reproducibility. This is particularly evident in MGMT methylation prediction, where inconsistent methodology and limited adherence to standardized reporting frameworks such as TRIPOD have yielded highly variable findings (35). The predominance of retrospective, single-center studies with small cohorts increases overfitting risk and limits external validation (35, 36, 70, 76). Most critically, continued reliance on MRI alone in many AI models, despite demonstrated PET complementarity, represents a significant missed opportunity for truly multimodal precision imaging (77).

When applying AI to multimodal imaging data, the limitations also involve its specific pitfalls, which extend beyond the already cited limitations. Other than small and retrospective cohort effects, AI literature is vulnerable to dataset bias, related to the acquisition protocols, often contextualized in academic contexts characterized by the presence of hybrid scanners, specific tracer protocols, and a standardized acquisition pipeline. Models trained on such data could fail in generalizing to institutions with different hardware, contrast agent protocols or even patient demographics, a distributional shift that is rarely assessed in the existing studies (85). A related concern is represented by algorithmic biases: advanced machine learning approaches based on deep learning architectures are extremely efficient in classifying, yet likewise difficult to explain and sensitive to factors that have little to no correlation to biological signals (slice thickness, scanner manufacturer, imaging-site effects) (63)). The adoption of explainability frameworks, such as SHAP (SHapley Additive explanations) (86) or Gradient-weighted class activation mapping (Grad-CAM) (87) can represent a strategy to address this issue, although their integration remains limited in GBM literature. Furthermore, the sensitivity of radiomic features to scanner manufacturer, magnetic field strength, and acquisition parameters implies that even the same lesion can differ substantially when observed in different institutions; imaging preprocessing steps, including normalization, resampling, bias field correction, and the techniques used to segment the tumor, may introduce additional sources of variability that can affect feature stability and biological signal itself. For this reason, following standardized definitions and computational protocols, such as the ones defined by Image Biomarker Standardization Initiative is becoming more and more crucial to improve cross-study comparability. Finally, Harmonization strategies, such as ComBat and its derivatives, represent powerful instruments to correct for site and scanner-wise batch effects in extracted features and have shown encouraging results (88).

Beyond the technical performance, the clinical adoption of AI-based imaging tools in GBM depends on several non-technical Factors that remain underdeveloped in current literature; factors such as differences in regulatory pathways across different jurisdictions, that typically require different analytical and clinical evidences of safety and reproducibility to approve a full implementation (89), and differences in model explainability, that despite some advancements, remains insufficient for routine neuro-oncological practice.

Moreover, prospective validation studies remain scarce, limiting confidence in generalizability, and cost-effectiveness analyses and formal implementation studies addressing infrastructure, personnel training, and reimbursement are largely absent from the GBM imaging AI literature, despite being essential to justify adoption within resource-constrained healthcare systems.

Finally, even technically and methodologically mature tools face applicational problems related to the expertise required to utilize them: the integration of complex AI-driven methodologies into existing clinical practice requires a fundamental shift in how specialized medical personnel interpret and act on imaging data (90). The adoption of AI as a clinically relevant support tool relies, when technical limitations are overcome, on system usability, liability framework, and ultimately the willingness of clinicians to incorporate this methodology in clinical practice (91, 92). For these reasons, future translational efforts must attend to interface usability, interpretability, and clinician education as to model performance.

Future research should prioritize prospective, multicenter studies built on harmonized protocols aligned with TRIPOD and IBSI standards (35), with attention to benchmark datasets, robustness to missing modalities, and explainability strategies clarifying which modality drives each prediction. Emphasis should be placed on genuinely multimodal AI frameworks integrating MRI and PET biomarkers validated against clinically meaningful endpoints.

Taken together, these findings indicate that progress in neuro-oncology AI will depend less on optimizing single-modality models and more on developing standardized multimodal frameworks integrating structural, functional, and metabolic data (62, 71, 83, 84). The most impactful systems will be those combining performance with transparency, robustness, and usability across heterogeneous clinical settings.
